# Toxic and Essential Elements in Rice and Other Grains from the United States and Other Countries

**DOI:** 10.3390/ijerph17218128

**Published:** 2020-11-03

**Authors:** Mom TatahMentan, Syprose Nyachoti, Laura Scott, Nati Phan, Frederick O. Okwori, Nedaa Felemban, Tewodros R. Godebo

**Affiliations:** Department of Environmental Health Sciences, School of Public Health and Tropical Medicine, Tulane University, New Orleans, LA 70112, USA; mtatahmentan@tulane.edu (M.T.); snyachoti@tulane.edu (S.N.); lscott9@tulane.edu (L.S.); nphan@tulane.edu (N.P.); ookwori@tulane.edu (F.O.O.); nfelemban@tulane.edu (N.F.)

**Keywords:** toxic and essential elements, rice and other grains, washed/unwashed rice, United States and other countries

## Abstract

We determined the concentrations of toxic and essential elements in rice and other grains (lentils, barleys, beans, oats, wheat, and peas) grown in the United States (US) and other countries using Inductively Coupled Plasma Mass Spectrometry (ICPMS). Results showed that median concentrations (in µg/kg) for toxic elements in white rice from the US were 131, 2.8, and 6.5 for arsenic (As), lead (Pb), and cadmium (Cd), respectively. White rice from Thailand, India, and Italy showed higher median toxic elements concentrations (in µg/kg) of 155 for As, 3.6 for Pb, and 8.4 for Cd, than for white rice from the US. Brown rice from the US showed median concentrations (in µg/kg) of 217 (As), 4.5 (Pb), and 17.4 (Cd) while other grains showed median concentrations (in µg/kg) of 5.4, 4.6, and 6.7 for these elements, respectively. None of the samples exceeded the codex standards set for Pb (200 μg/kg in cereals and pulses) and Cd (100 μg/kg in cereals/pulses and 400 μg/kg in polished rice). However, brown rice and one white rice sample did exceed the codex standard for As (200 μg/kg). Essential elements were higher in other grains than in white and brown rice. These findings suggest that alternating or coupling rice with other grains in one’s diet could reduce exposure to toxic metals while providing more essential elements to rice diet.

## 1. Introduction

Toxic metals primarily originating from industrialization have polluted the environment (e.g., soils, air, water, and food) and caused adverse human health effects through food chains [1]. Exposure to low and high levels of arsenic (As), lead (Pb), and cadmium (Cd) can cause cancerous and non-cancerous effects [2,3,4]. For example, As toxicity has been attributed to skin, lung, kidney, and bladder cancers [5]. Cadmium and Pb toxicity have been linked to lung, prostate, and kidney cancers [5]. Human exposure to these toxic metals can occur through occupation, air pollution, or diet. Dietary exposure is the most common route through which these toxic metals enter the human body [6]. Grains such as rice, maize, wheat, beans, oats, lentils, and peas are a major part of the daily diet that provides carbohydrates, proteins, and other nutrients to people around the world. These cereals and legumes may contain toxic metals, which the human body could be exposed to through ingestion. For example, rice is known to accumulate more metals than other cereals [7,8,9]. As a staple food, particularly in the Asian populations, rice could be a major source of toxic metals intake [8]. Toxic metals are both naturally occurring and/or introduced through anthropogenic activities into soils where the crops are grown [10]. The metals are absorbed and accumulated in the edible plant parts and enters the food chain. The Codex Alimentarius Commission has set maximum permissible levels for these elements in various grains to protect trade and health [11]. Because of the daily need to nourish our bodies, evaluation of toxic metal exposure through consumption of staple cereal grains and legumes need attention.

About 85% of rice in the US is locally produced while the rest is imported from Asia, including Thailand, India, and Pakistan. Rice in the US is mainly produced in the southern states (Arkansas, Louisiana, Mississippi, Missouri, and Texas), which accounts for 80% of the production while California state produces 20% [12]. Short and medium grain rice types are mainly grown in California for domestic and export purposes whereas the southern states primarily produce the long grain rice [12]. 

Concentrations of toxic elements in grains from different countries have been determined previously [8,13,14,15]. Arsenic concentrations in rice from the US and France were found to be higher than other countries [13]. Studies have shown that As concentrations in rice differ by state within the US, suggesting varying metal contamination in the environments; for example, rice produced in Texas and Arkansas contained more As than rice grown in California [15]. Louisiana is among the states that produce most of the rice consumed in the US. However, few studies have determined toxic element concentrations of rice grown or consumed in Louisiana [16]. This study aims to determine the concentrations of toxic metals and essential elements in brown and white rice as well as lentils, barleys, beans, oats, wheat, and peas (here after called other grains), grown in the US and other countries. We also assessed the effect of washing on the variation of toxic and essential metal concentrations in white rice. Concentrations of essential and toxic elements in rice and other cereals due to geographical locations allow us to relate the metal toxicity in the grains, and the potential health risks.

## 2. Methods

### 2.1. Sample Collection, Preparation, and Analysis

A total of 28 white and 11 brown rice samples were purchased from various local stores in Louisiana, US. Based on the package label information, the white rice samples were grown in the US (California, Texas, and Louisiana), Thailand, Italy, and India. All brown rice samples were produced in Louisiana, Arkansas, and Texas. Other grains purchased from various local stores in Louisiana included lentils, barleys, beans, oats, wheat, and pea samples. These grains were grown in Canada and various states in the US. 

A portion (~5 g) of each sample was powdered using a pestle and mortar. A modified wet sample digestion method of Akinyele and Shokunbi [17] was adopted. Briefly, about 0.1 g of each sample was weighed into acid washed Teflon digestive beakers separately. An aliquot of high-purity acids, including 3 mL of 67–69% Fisher Optima nitric acid and 0.5 mL of 32–35% Optima hydrochloric acid, were added. The mixtures were allowed to stand for three hours at room temperature, and then placed on a hotplate overnight at 75 °C. The samples were degassed, and 0.5 mL of pure hydrogen peroxide was added before heating them at 100 °C overnight. The samples were then cooled and degassed, and then 0.5 mL ultra-pure distilled water was added to each vessel and placed on the hot plate overnight at 100 °C. The samples were digested completely until a clear solution appeared for analysis using Inductively Coupled Plasma Mass Spectrometry (Agilent 7900 ICP-MS). For the rinse washing, nine randomly selected white rice samples were rinsed three times with ultra-pure distilled water until the water became clear to remove any external contamination (e.g., dirt and dust), to represent rice-rinsing practices in a household. The washed samples were then dried at 80 °C for three hours, powdered, and analyzed using procedures used for the unwashed samples. We analyzed the standard reference materials (Rice, NIST 1568b) purchased from the National Institute of Standards and Technology (NIST) to verify the accuracy of our procedures. The mean recoveries of the toxic and essential elements in NIST 1568b ranged from 78 to 110%.

### 2.2. Statistical Analyses

All statistical analyses were performed using GraphPad Prism version 8.2.1 software. Descriptive analyses included means, standard deviation, and quartiles of quantitative variables. Kruskal–Wallis and Mann–Whitney tests were conducted to compare the means, and the significance level was considered at *p* < 0.05.

## 3. Results

Toxic and essential elements concentrations (median (25th–75th percentile) in rice samples and other grains are shown in Table 1. Percentage elemental loss from rinse-washed white rice is presented in Table 2.

### 3.1. Toxic Elements Concentrations in Rice and Other Grains

**Figure 1:**
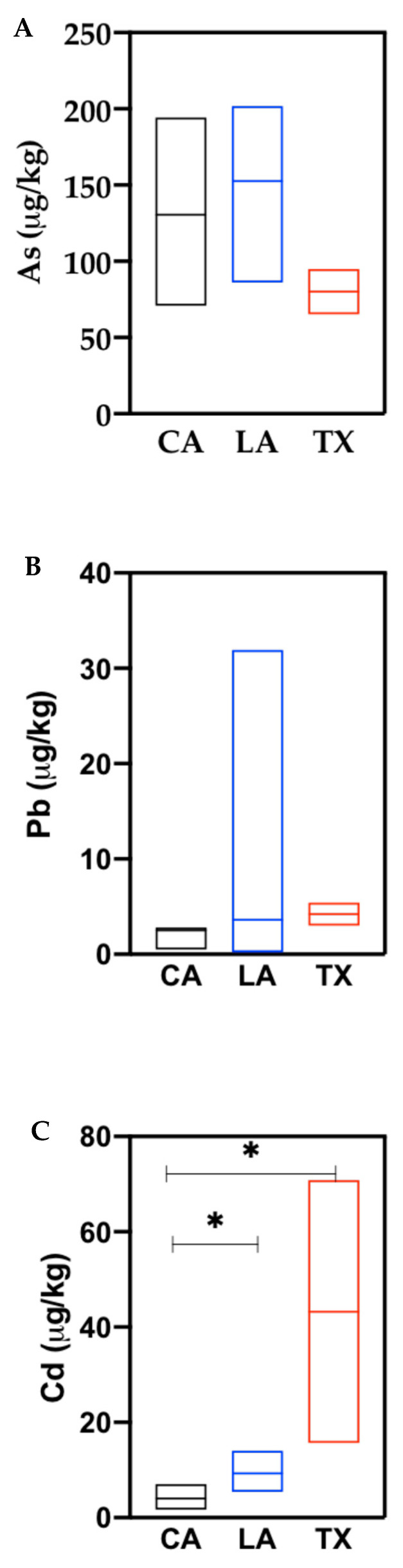
Toxic elements (As, Pb, and Cd) concentrations in white rice from the US (corresponding to Figure 1A, Figure 1B and Figure 1C respectively); the lines in the plots show median concentrations. Statistically significant (* *p* < 0.05) concentrations are based on non-parametric tests of the means.

**Figure 2:**
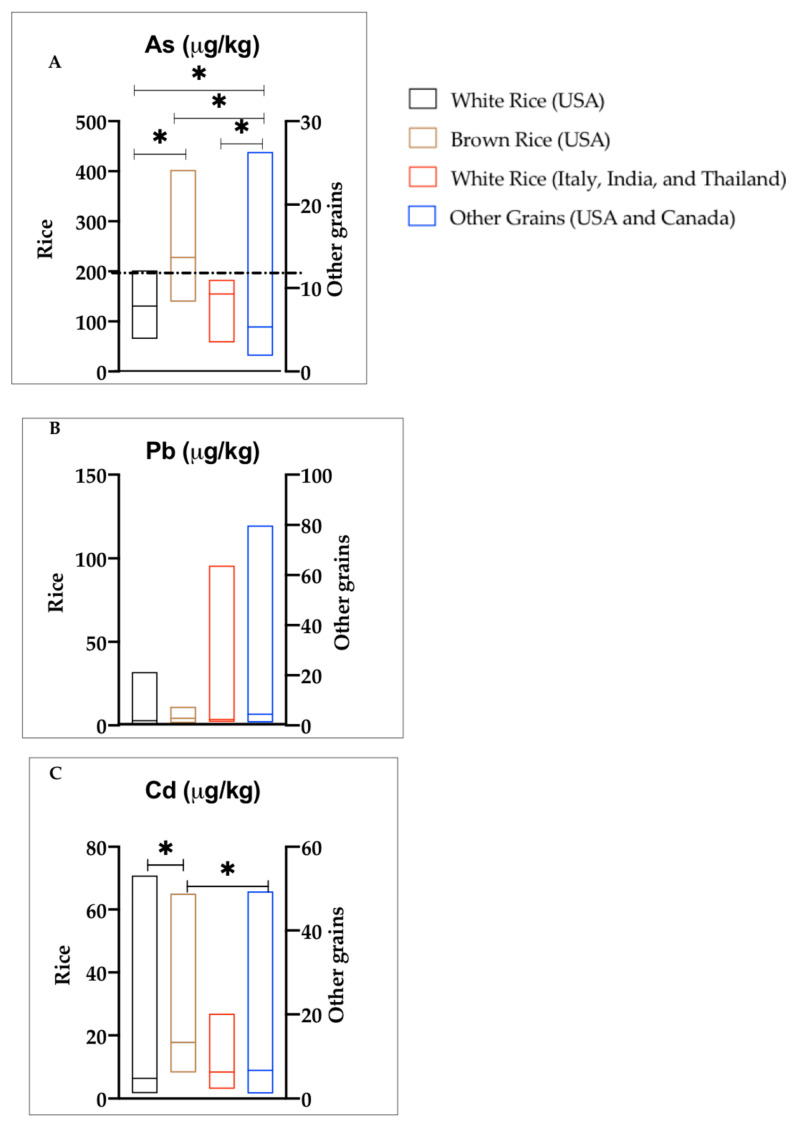
Toxic elements (As, Pb, and Cd) concentrations in rice and other grains (corresponding to Figure 2A, Figure 2B and Figure 2C respectively); the lines in the plots show median concentrations. Statistically significant (* *p* < 0.05) concentrations are based on non-parametric tests of the means. The dotted line at 200 μg/kg in figure A indicates the codex maximum limit for As in rice. Other elements were below the codex standards set for Pb (200 μg/kg in cereal grains and pulses) and Cd (100 μg/kg in cereals/pulses and 400 μg/kg in polished rice).

The median (25th–75th percentile (in µg/kg)) concentrations of toxic metals in white rice from the US were 131 (90–157), 2.8 (2.4–5), and 6.5 (4.7–12) for As, Pb, and Cd, respectively (Table 1). The highest concentrations (in µg/kg) of As (202) and Pb (32) in white rice were from Louisiana, while white rice from Texas had the lowest As (65) (Figure 1A,B). Highest Cd concentrations in white rice were from Texas (71 µg/kg), while the lowest Cd levels were measured in California (1.7 µg/kg) states. Mean cadmium concentrations in white rice from California were significantly different from those of Louisiana and Texas states (Figure 1C). Arsenic, Pb, and Cd median concentrations (in µg/kg) in white rice originally from Thailand, India, and Italy were As 155 (93–167), Pb 3.6 (2.5–7.6), and Cd 8.4 (5.1–17). White rice from Thailand, India, and Italy had higher median concentrations of these elements compared to white rice from US (Table 1). Brown rice from the US had median concentrations (in µg/kg) at 217 (180–291) for As, 4.5 (2.6–11) for Pb, and 17.4 (9.5–42) for Cd (Table 1). The highest As, Pb, and Cd in brown rice were from Texas; however, there were no significant differences in mean concentrations of toxic metals in the considered states. Arsenic and Cd mean concentrations in white rice from the US were significantly different from brown rice (US) (Figure 2A,C). Other grains had median concentrations (in µg/kg) of 5.4 (3.2–9.1), 4.6 (3.5–8), and 6.7 (2.6–49) for As, Pb, and Cd, respectively (Table 1). All median concentrations of toxic metals were higher in the white and brown rice samples than in other grains; however, significant differences were observed in mean As concentrations between rice (white and brown) and other grains only (Figure 2A). 

### 3.2. Selected Essential Elements Concentrations in Rice and Other Grains

White rice from the US had median concentrations of 261, 41, 680, 3, 10, 2.5, and 12 for Mg, Ca, K, Fe, Mn, Cu, and Zn, respectively. White rice from Italy, India, and Thailand showed median concentrations (in mg/kg) of 123 for Mg, 43 for Ca, 574 for K, 2 for Fe, 8 for Mn, 1.77 for Cu, and 13 for Zn (Table 1). Brown rice (US) showed median concentrations (in mg/kg) of 1240 for Mg, 86 for Ca, 2355 for K, 12 for Fe, 30 for Mn, 3.5 for Cu, and 18 for Zn (Table 1). The median concentrations (in mg/kg) of essential elements in other grain samples were 1090, 455, 8894, 60, 14, 8.03, and 29 for Mg, Ca, K, Fe, Mn, Cu, and Zn, respectively (Table 1). All median concentrations for essential elements were higher in other grains except Mg and Mn, which accumulated more in brown rice. 

## 4. Discussion

### 4.1. Toxic Elements in Rice and Other Grains

Arsenic, Pb, and Cd are among the toxic metals that pose serious health effects in humans. Exposure to these metals through diet is of concern, especially among rice consumers. Essential elements in food such as Ca, Mg, Zn, Fe, Cu, Mn, Co, and Sr support the various physiological body functions, such as bone regeneration, enzyme activities, and synthesis of proteins [18].

#### 4.1.1. Arsenic

Generally, both white and brown rice had higher median concentration of As than other grains (Table 1). There was a significant difference between mean As concentrations in white rice (US), brown rice (US), white rice (Italy, India, and Thailand), and other grains (Figure 2A). This is consistent with previous studies, which have reported that rice absorbs more As than other grains such as wheat and barley due to higher transfer rates of As from soil to grain [7]. Moreover, rice is a staple food that contributes toxic, inorganic As to humans through diet [19,20]. Brown rice accumulated more As than white rice; this is likely because brown rice has a germ layer that retains greater amounts of inorganic As [21]. Highest As concentrations were measured in white rice from Louisiana, whereas the lowest concentrations were from Texas states. However, no significant difference was observed in mean As concentrations in white rice from different states in the US. Approximately two billion people in Asia rely on rice as a staple food [22]. Rice and other foods may increase As exposure among the Asian population. We report higher median As concentrations in white rice (Italy, India, and Thailand) (155 μg/kg) than in white rice (US) (131 μg/kg) (Table 1). Median As content in white rice (Italy, India, and Thailand) of this study falls within the range of values reported in other works. For example, market survey studies have reported mean As concentrations (in μg/kg) ranging from 70–310, 60–500, and 190–220 in India, Thailand, and Italy, respectively [13,14,23]. On the contrary, Meharg et al. [13] reported higher total As content in white rice from the US than in white rice from India, Italy, and Thailand. We did not find any significant difference in mean As concentrations between white rice from the US and white rice (Italy, India, and Thailand) (Figure 2A). However, we report high As concentrations (202 μg/kg) in white long grain rice from the US, which is in the range of values for mean As concentrations of 200–460 μg/kg previously reported in white rice from the US [14,19,24]. The Codex Alimentarius Commission has set the maximum level of inorganic arsenic in rice to be 200 μg/kg for trade and health protection [11]. Six brown rice samples and one white, long grain rice from the US exceeded this limit. However, health risks associated with consumption of As-contaminated foods depend on the amount of As consumed daily, its form, and its bioavailability.

#### 4.1.2. Lead

White rice (US) had lower median Pb concentrations (in μg/kg) of 2.8 than brown rice (4.5), both of which were lower than other grains (4.6) (Table 1). By comparison, Salama [25] reported mean Pb concentrations (in μg/kg) of 13–24, 131, 398, and 239 in whole and seed-split lentils, barley, wheat, and rice sampled from Egyptian markets, respectively. These concentrations are much higher than the median Pb concentrations of other grains and rice reported in this study. Median Pb concentrations in white rice (US) in our study are lower than the mean values reported in Norton et al. [26] (11 μg/kg) and Adomako et al. [23] (8 μg/kg) in market-surveyed white rice. However, we show that brown rice accumulated more Pb than white rice. This is consistent with the works of Norton et al. [26], who reported higher mean Pb concentrations (21 μg/kg) in field-collected brown rice and lower mean Pb levels (11 μg/kg) in US-market-collected white rice. White rice (Italy, India, and Thailand) had the highest mean concentration of lead (14 μg/kg, Table 1), which is consistent with values (3–11 μg/kg) reported in Adomako et al. [23] and Norton et al. [26] in market-surveyed white rice from Italy, India, and Thailand. However, Norton et al. [26] reported higher mean Pb concentrations (23 μg/kg) in market-collected white rice from India. Their work shows that on a global scale Indian white rice is the fourth most abundant in Pb concentrations (in μg/kg) after China (46–185), Nepal (33), and Ghana (24). All samples analyzed in this study were below the Codex standards limit of 200 µg/kg for Pb in cereals (including for pulses and legumes) [11].

#### 4.1.3. Cadmium

Similar to As, brown rice from the US in our study had higher median Cd concentrations (17.4 μg/kg) than white rice from the US (6.5 μg/kg) and white rice from Italy, India, and Thailand (8.4 μg/kg). There was a significant difference in mean concentrations between brown rice and white rice, as well as other grains (Figure 2C). Like As, Cd has also been found to accumulate more in rice than other cereal grains [8,27]. Brown rice contained more Cd than white rice, likely because the grain contains an outer layer, which accumulates metals more easily [21]. Meharg et al. [8] has shown that milling of rice reduces the levels of Cd in the grain. We found low median levels of Cd in white rice (US) and white rice (Italy, India, and Thailand) than values in literature. For example, both Adomako et al. [23] and Meharg et al. [8] reported slightly higher mean Cd concentrations (18 μg/kg) in white rice from the US. Within the US, white rice from California and Louisiana had Cd concentrations ranging from 1.7 to 14 μg/kg; the highest Cd levels (70 μg/kg) were reported in white rice from Texas (Figure 1C). White rice from Italy, India, and Thailand showed mean values ranging from 27 to 78 μg/kg [8], while Adomako et al. [23] reported mean values ranging from 18 to 21 μg/kg in white rice from these three countries. Despite the data discrepancies, Meharg et al. [8] classified Cd concentrations from the US, Italy, India, and Thailand as intermediate. Indeed, Cd concentrations of this study and those reported in Adomako et al. [23] and Meharg et al. [8] are much lower than the mean concentration (240 μg/kg) reported in unpolished rice irrigated with untreated mining wastewater in China [28]. None of the samples in our study exceeded the Codex standards for Cd in cereal grains/pulses (100 μg/kg), polished rice (400 μg/kg), and wheat (200 μg/kg) [11].

### 4.2. Essential Elements in Rice and Other Grains

Median concentrations of Ca, K, Fe, Cu, and Zn were higher in other grain samples than in brown and white rice; however, brown rice had the highest Mg and Mn (Table 1). Rice is a commonly consumed cereal throughout the world [29]. Though rice and other cereals in general may provide essential minerals such as Ca, Mg, Fe, Zn, Cu, and Mn to humans; presence of other compounds such as phytic acid in whole grains; and subsequent chelation of these elements may limit their bioavailability [30]. Alternatively, other grains, including pulses, provide significant essential elements such as Zn, Ca, and K [30]. Thompson et al. [31] suggest that incorporating cereals (such as rice) with other grains (e.g., beans) provides improved nutritional health effects compared to consuming rice alone.

### 4.3. Effect of Washing Process on the Concentrations of Toxic and Essential Elements in White Rice

Washing of white rice to remove any external contamination before analyses reduced concentrations of toxic elements such as Pb and Cd by 57% and 46%, respectively (Table 2). Washing of rice is accompanied by loss of essential elements that aid normal body functioning. The concentrations of essential elements reduced by 51% for Ca, 74% for Mg, 43% for K, 74% for Fe, and 8.3% for Zn (Table 2). Our findings are consistent with the works of Horner and Beauchemin [32], who reported that washing rice with double deionized water before cooking led to a great loss of essential elements, including Cu, Fe, and Zn. Such a decrease in essential nutrients may lead to nutrient deficiency in populations consuming rice as staple food, especially young children and pregnant women. More work is needed to optimize rice-to-water ratios in the washing process in order to minimize significant loss of essential nutrients in washed rice.

## 5. Conclusions

Toxic metal exposure through diet is a public health concern. As a result, food safety is an issue that threatens human health and agricultural trade. This study assessed the distribution of toxic and essential elements in rice and other grains from different geographical locations (USA, Italy, India, Thailand, and Canada) consumed in the US. The study also assessed the effect of rinse washing on concentrations of toxic and essential elements in white rice. White rice from Thailand, India, and Italy showed higher median concentrations of toxic metal such as As, Pb, and Cd compared to white rice from the US. Lead and Cd concentrations did not exceed the codex standards; however, As concentrations in brown rice and one white rice from US exceeded the codex standards. White and brown rice had higher median concentrations of toxic metals than other grains, which in turn had higher median concentrations of essential elements. Our study shows that coupling rice with other grains in a meal could provide additional essential elements, which could be insufficient rice diet. We also showed that washing of rice reduces some toxic and essential elements in rice. Elemental distribution in grains from different regions will help countries to make informed decisions on importing cereals such as rice. Rice and other grain producers can establish strategies to reduce significant metal uptake from soils. Identifying proper rice treatment processes such as washing provides the rice-consuming population with information on reducing metal exposure while still conserving essential elements in the grain.

## Figures and Tables

**Table 1:** Toxic and essential elements concentrations in rice and other grains.

Sample	White Rice (US)				White Rice (Italy, India, Thailand)				Brown Rice				Other Grains			
		Percentile				Percentile				Percentile				Percentile		
μg/kg (Dry Weight)	Mean (Min–Max)	25th	50th	75th	Mean (Min–Max)	25th	50th	75th	Mean (Min–Max)	25th	50th	75th	Mean (Min–Max)	25th	50th	75th
As	129	90.3	131	157	136	93	155	167	243	180	217	291	7.6	3.2	5.4	9.1
	(65–202)				(58–183)				(139–403)				(1.9–26)			
Pb	5.6	2.4	2.8	5	14	2.5	3.6	7.6	7.4	2.6	4.5	11	9.7	3.5	4.6	8
	(0.2–32)				(2–96)				(1.4–34)				(1.2–80)			
Cd	11	4.7	6.5	12	12	5.1	8.4	17	24	9.5	17.4	42	11	2.6	6.7	49
	(1.7–71)				(3.1–27)				(7.7–65)				(1.2–49)			
**Mg/kg** **(Dry weight)**																
Mg	260	98	261	314	140	78	123	212	1220	1100	1240	1340	1190	967	1090	1430
	(55–1110)				(52–266)				(937–1410)				(490–2090)			
Ca	57	36	41	65	61	34	43	56	87	74	86	96	520	294	455	603
	(24–234)				(23–222)				(64–116)				(135–2073)			
K	833	575	680	932	624	497	574	742	2413	2155	2355	2731	7915	4035	8894	9910
	(270–2252)				(477–891)				(1981–3003)				(2157–15986)			
Fe	4.3	2	3	7	2.3	2	2	2.5	12	10	12	14	55	38.7	60	53.8
	(1–11)				(1–4)				(7–16)				(23–114)			
Mn	11	8	10	12	8.8	7	8	10.5	29.2	26	30	33	19	12	14	25.6
	(5–27)				(7–13)				(18–35)				(8–42)			
Cu	2.67	2.2	2.5	2.69	2.48	1.61	1.77	3.84	3.43	2.87	3.45	3.9	7.8	5.29	8.03	10.1
	(1.8–5.2)				(1.39–4.43)				(2.24–4.77)				(3.64–12.1)			
Zn	12.5	11.3	12	14.8	13.2	12	13	14.3	18.2	16	18	20.5	28	21.5	29	32
	(8–19)				(12–15)				(15–23)				(19–40)			

**Table 2:** Percentage elemental loss of toxic and essential elements from rinse-washed white rice.

Sample	Washed White Rice (9)	Unwashed White Rice (19)	% Elemental Loss from White Rice
	Mean (μg/kg, Dry Weight)	Mean (μg/kg, Dry Weight)	Percentage (%)
Pb	4.3	10	57
Cd	7.0	13	46
	Mean (mg/kg, Dry Weight)	Mean (mg/kg, Dry Weight)	Percentage (%)
Ca	34	69.3	50.9
Mg	75.6	290	73.9
K	511	887	42.5
Fe	1.20	4.7	74.3
Mn	7.99	11.2	28.4
Zn	12.1	13.2	8.25

Note: Numbers in brackets indicate number of samples analyzed.
